# Presentation and outcomes of attention deficit and hyperactivity disorder in females and males

**DOI:** 10.1192/j.eurpsy.2021.224

**Published:** 2021-08-13

**Authors:** O. Kilic, S. Young

**Affiliations:** 1 Department Of Psychiatry, Bezmialem Vakif University Medical Faculty, Istanbul, Turkey; 2 Psychology, Psychology Services Limited, London, United Kingdom; 3 Department Of Psychology, Reykjavik University, Reykjavik, Iceland

## Abstract

**Abstract Body:**

Attention deficit and hyperactivity disorder (ADHD) is a common neurodevelopmental disorder in children. Its primary clinical features include symptoms of inattention and hyperactivity/impulsivity, although young people often present with emotional dysregulation, excessive mind-wandering and executive dysfunction. Symptoms of ADHD often persist into adulthood together with high rates of comorbidity and significant psychosocial impairment across the lifespan. Berry, Shaywitz and Shaywitz proposed over 30 years ago that girls with ADHD form a ‘silent minority’ with greater internalized behavior which leads them to be under-identified. Even when referred for clinical assessment, their ADHD symptoms are missed or misdiagnosed for other conditions such as anxiety, depression and personality disorder. This means they will not receive the treatment they need. Compared with controls, they may be especially vulnerable to childhood adversities and health problems and they may cope with these difficulties with dysfunctional strategies (eg. with substance misuse and/or deliberate self-harming behaviours). If we are to enhance long-term outcomes in girls and women with ADHD, healthcare practitioners need to better understand the presentation of ADHD in females, improve detection and assessment of ADHD in order that they may access appropriate treatment. This workshop will focus on the differences in presentation and outcomes between males and females with ADHD.

**Disclosure:**

No significant relationships.

